# Supplementary material for: Once upon a time in Mexico: Holocene biogeography of the spotted bat (Euderma maculatum)
Source: PLoS One. 2023 May 10;18(5):e0274342. doi: 10.1371/journal.pone.0274342 (PMC10171611; doi:10.1371/journal.pone.0274342)
Supplement: S1 File — (PDF) [file pone.0274342.s001.pdf]

S1 Table 1. List of *Euderma maculatum* occurrences retrieved for ecological niche modeling. Geographic coordinates were retrieved from Vertnet (vertnet), Arctos (museum), or from field capture (field). Geographic coordinates labeled "Obscured" indicate a single sensitive locality and are obscured at the request of land managers.

Specimen/Occurrence	Latitude	Longitude	Type	Collection year	State/Province	Country
3_2009_Cave_M	Obscured	Obscured	field	2009	Arizona	United States
4_2009_Cave_M	Obscured	Obscured	field	2009	Arizona	United States
5_2009_Cave_M	Obscured	Obscured	field	2009	Arizona	United States
6_2009_Cave_M	Obscured	Obscured	field	2009	Arizona	United States
8_2009_Cave_M	Obscured	Obscured	field	2009	Arizona	United States
10_2009_Cave_M	Obscured	Obscured	field	2009	Arizona	United States
12_2009_Cave_M	Obscured	Obscured	field	2009	Arizona	United States
16_2009_Cave_M	Obscured	Obscured	field	2009	Arizona	United States
18_2009_Cave_M	Obscured	Obscured	field	2009	Arizona	United States
19_2009_Cave_M	Obscured	Obscured	field	2009	Arizona	United States
47_2009_Cave_M	Obscured	Obscured	field	2009	Arizona	United States
75_2009_Cave_M	Obscured	Obscured	field	2009	Arizona	United States
76_2009_Cave_M	Obscured	Obscured	field	2009	Arizona	United States
N11_2009_Cave_M	Obscured	Obscured	field	2009	Arizona	United States
N2_2009_Cave_M	Obscured	Obscured	field	2009	Arizona	United States
N3_2009_Cave_M	Obscured	Obscured	field	2009	Arizona	United States
Bat4_2010_Cave_M	Obscured	Obscured	field	2010	Arizona	United States
Bat5_2010_Cave_F	Obscured	Obscured	field	2010	Arizona	United States
Bat6_2010_Cave_F	Obscured	Obscured	field	2010	Arizona	United States
Bat7_2010_Cave_F	Obscured	Obscured	field	2010	Arizona	United States
Bat8_2010_Cave_M	Obscured	Obscured	field	2010	Arizona	United States
Bat9_2010_Cave_F	Obscured	Obscured	field	2010	Arizona	United States
Bat10_2010_Cave_F	Obscured	Obscured	field	2010	Arizona	United States
Bat11_2010_Cave_M	Obscured	Obscured	field	2010	Arizona	United States
Bat12_2010_Cave_M	Obscured	Obscured	field	2010	Arizona	United States
Bat13_2010_Cave_M	Obscured	Obscured	field	2010	Arizona	United States
Bat14_2010_Cave_M	Obscured	Obscured	field	2010	Arizona	United States
Bat26_M2010_Cave_M	Obscured	Obscured	field	2010	Arizona	United States
201_2013_Cave_F	Obscured	Obscured	field	2013	Arizona	United States
202_2013_Cave_F	Obscured	Obscured	field	2013	Arizona	United States
203_2013_Cave_F	Obscured	Obscured	field	2013	Arizona	United States
204_2013_Cave_M	Obscured	Obscured	field	2013	Arizona	United States
205_2013_Cave_M	Obscured	Obscured	field	2013	Arizona	United States

Specimen/Occurrence	Latitude	Longitude	Type	Collection year	State/Province	Country
206_2013_Cave_M	Obscured	Obscured	field	2013	Arizona	United States
2014 Cave 3	Obscured	Obscured	field	2014	Arizona	United States
2014 Cave 5	Obscured	Obscured	field	2014	Arizona	United States
Bat21_2010_V_F	36.634139	-111.94593	field	2010	Arizona	United States
Bat22_2010_V_M	36.634139	-111.94593	field	2010	Arizona	United States
210_2013_V_M	36.634139	-111.94593	field	2013	Arizona	United States
211_2013_V_F	36.634139	-111.94593	field	2013	Arizona	United States
212_2013_V_F	36.634139	-111.94593	field	2013	Arizona	United States
Bat15_2010_NR_M	36.690263	-112.28236	field	2010	Arizona	United States
Bat16_2010_NR_M	36.620282	-112.29583	field	2010	Arizona	United States
Bat20_2010_NR_F	36.646129	-112.31206	field	2010	Arizona	United States
Bat23_2010_NR_F	36.481963	-112.29835	field	2010	Arizona	United States
Bat24_2010_NR_F	36.524693	-112.37937	field	2010	Arizona	United States
Bat25_2010_NR_F	36.524693	-112.37937	field	2010	Arizona	United States
207_2013_NR_M	36.617077	-112.21943	field	2013	Arizona	United States
208_2013_NR_M	36.617077	-112.21943	field	2013	Arizona	United States
209_2013_NR_F	36.617077	-112.21943	field	2013	Arizona	United States
Bat1_2009_SR_F	35.882297	-111.94073	field	2009	Arizona	United States
200_2013_SR_F	35.873765	-111.89847	field	2013	Arizona	United States
140623_1_F_NM	32.706126	-105.7979	field	2014	New Mexico	United States
140721_19_F_CACH_AZ	36.087946	-109.3863	field	2014	Arizona	United States
140721_22_F_CACH_AZ	36.087946	-109.3863	field	2014	Arizona	United States
140722_41_F_CACH_AZ	36.128785	-109.465	field	2014	Arizona	United States
140804_03_F_Canada	50.825377	-121.86815	field	2014	British Columbia	Canada
140818_022_M_Canada	50.825377	-121.86815	field	2014	British Columbia	Canada
140818_033_F_Canada	50.825377	-121.86815	field	2014	British Columbia	Canada
140818_04_M_Canada	50.825377	-121.86815	field	2014	British Columbia	Canada
140818_05_F_Canada	50.825377	-121.86815	field	2014	British Columbia	Canada
140818_06_F_Canada	50.825377	-121.86815	field	2014	British Columbia	Canada
140818_07_F_Canada	50.825377	-121.86815	field	2014	British Columbia	Canada
2014 Cave 1	Obscured	Obscured	field	2014	Arizona	United States
2014 Cave 2	Obscured	Obscured	field	2014	Arizona	United States
2014 Cave 4	Obscured	Obscured	field	2014	Arizona	United States
KU:119275	29.25	-103.25	vertnet/museum	1968	Texas	United States
KU:139030	37.07	-113.57	vertnet/museum	1983	Utah	United States
LACM:009822	35.72361	-117.61361	vertnet/museum	1956	California	United States
LACM:009832	37.80278	-114.41222	vertnet/museum	1948	Nevada	United States
LACM:13855	26.699994	-106.13641	vertnet/museum	1961	Chihuahua	Mexico
LACM:13856	26.699994	-106.13641	vertnet/museum	1961	Chihuahua	Mexico

Specimen/Occurrence	Latitude	Longitude	Type	Collection year	State/Province	Country
LACM:9823	33.4147	-111.9086	vertnet/museum	1953	Arizona	United States
MCZ:42130	32.6697	-114.624	vertnet/museum	1904	Arizona	United States
MSB:MAMM:107557	40.80944	-108.94833	vertnet/museum	1981	Colorado	United States
MSB:MAMM:112056	40.33	-108.82	vertnet/museum	1987	Colorado	United States
MSB:MAMM:112057	40.33	-108.82	vertnet/museum	1987	Colorado	United States
MSB:MAMM:114512	44.95309	-108.158	vertnet/museum	1990	Wyoming	United States
MSB:MAMM:114513	44.95309	-108.158	vertnet/museum	1990	Wyoming	United States
MSB:MAMM:116832	38.28278	-111.24778	vertnet/museum	1988	Utah	United States
MSB:MAMM:17285	33.3455012	-108.7064	vertnet/museum	1963	New Mexico	United States
MSB:MAMM:23376	35.8200981	-106.5918	vertnet/museum	1966	New Mexico	United States
MSB:MAMM:23378	35.8200981	-106.5918	vertnet/museum	1966	New Mexico	United States
MSB:MAMM:24999	33.575781	-107.37791	vertnet/museum	1967	New Mexico	United States
MSB:MAMM:25000	33.575781	-107.37791	vertnet/museum	1967	New Mexico	United States
MSB:MAMM:27715	35.8200981	-106.5918	vertnet/museum	1967	New Mexico	United States
MSB:MAMM:37688	35.241607	-107.60874	vertnet/museum	1978	New Mexico	United States
MSB:MAMM:37724	33.5575983	-107.3923	vertnet/museum	1978	New Mexico	United States
MSB:MAMM:6235	36.325001	-106.50445	vertnet/museum	1958	New Mexico	United States
MSB:MAMM:9606	33.3972145	-108.63839	vertnet/museum	1960	New Mexico	United States
MSB:MAMM:9608	33.3972145	-108.63839	vertnet/museum	1960	New Mexico	United States
MSB:MAMM:9609	33.3972145	-108.63839	vertnet/museum	1960	New Mexico	United States
MSB:MAMM:9610	33.3972145	-108.63839	vertnet/museum	1960	New Mexico	United States
MSB:MAMM:121373	37.604161	-110.02986	vertnet/museum	1994	Utah	United States
MSB:MAMM:127230	40.756372	-108.88219	vertnet/museum	1994	Colorado	United States
MSB:MAMM:135536	35.109983	-106.63973	vertnet/museum	1994	New Mexico	United States
MSB:MAMM:23376	35.8200981	-106.5918	vertnet/museum	1966	New Mexico	United States
MVZ:MAMM:139209	37.749459	-119.58679	vertnet/museum	1931	California	United States
NMMNH:MAMM:ASK0692	20.816028	-99.721286	vertnet/museum	1984	Queretaro	Mexico
NMMNH:MAMM:1901	33.722817	-107.46246	vertnet/museum	1992	New Mexico	United States
NMMNH:MAMM:4059	36.71382	-114.62783	vertnet/museum	2000	Nevada	United States
RBCM:10799	49.184	-119.5479	vertnet/museum	1980	British Columbia	Canada
TCWC:26538	20.61	-100.384	vertnet/museum	1972	Queretaro	Mexico
TSM:306333	47.252878	-122.44429	vertnet/museum	2013	Washington	United States
UCM:15179	36.991	-113.57191	vertnet/museum	1974	Utah	United States
UCM:15180	37.009106	-113.49584	vertnet/museum	1974	Utah	United States
UCM:15181	37.009106	-113.49584	vertnet/museum	1974	Utah	United States
UCM:7335	36.852	-107.995	vertnet/museum	1960	New Mexico	United States
UMNH:1135	40.65213	-111.93299	vertnet/museum	1934	Utah	United States
UMNH:30395	38.31	-111.37	vertnet/museum	2001	Utah	United States
USNM:269842	32.753336	-113.83139	vertnet/museum	1940	Arizona	United States

Specimen/Occurrence	Latitude	Longitude	Type	Collection year	State/Province	Country
UWBM:MAMM:82236	44.6056	-121.277	vertnet/museum	2012	Oregon	United States
UWBM:MAMM:82237	44.6056	-121.277	vertnet/museum	2012	Oregon	United States

S1 Table 2. List of dereplicated geographic coordinates (1 km^2) of *Euderma maculatum* that were used for training Maxent models.

Dereplicated occurrence	Longitude	Latitude
1	-107.9375	45.3125
2	-108.92083	40.7958333
3	-108.8875	40.7291667
4	-108.9875	40.6041667
5	-108.67083	40.4541667
6	-108.84583	39.2291667
7	-111.2375	38.4458333
8	-111.24583	38.2791667
9	-110.02917	37.6041667
10	-117.6625	37.4208333
11	-118.39583	37.3625
12	-108.4125	37.2791667
13	-113.57083	37.0708333
14	-113.4875	37.0041667
15	-118.95417	36.7375
16	-112.27917	36.6875
17	-112.3125	36.6458333
18	-111.94583	36.6375
19	-112.29583	36.6208333
20	-112.22083	36.6208333
21	-111.7375	36.6125
22	-112.37917	36.5208333
23	-112.29583	36.4791667
24	-111.89583	36.4708333
25	-109.4625	36.1291667
26	-109.3875	36.0875
27	-111.9375	35.8791667
28	-111.89583	35.8708333
29	-106.59583	35.8208333
30	-107.67083	35.2458333
31	-107.6125	35.2375
32	-106.64583	35.1125

Dereplicated occurrence	Longitude	Latitude
33	-106.6375	35.0708333
34	-106.8625	33.9208333
35	-107.39583	33.5541667
36	-105.69583	32.7125
37	-105.79583	32.7041667
38	-103.24583	29.2458333
39	-103.6125	29.2291667
40	-106.1375	26.6958333

S1 Fig 1. Cropped background extent and dereplicated points (1 km^2) used for the training ecological niche models. The shaded region (grey) gives the spatial extent for which background points were randomly sampled for model training.

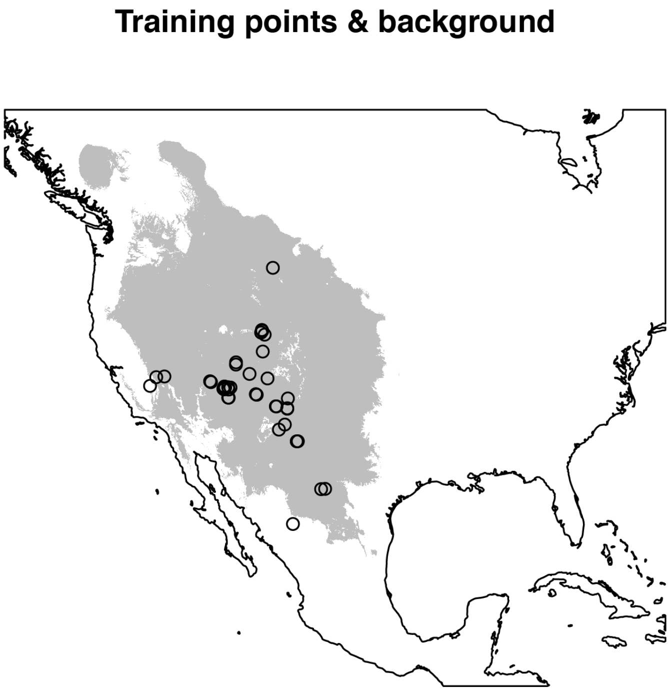

S1 Fig 2. Continuous range map projections from Maxent ENM illustrating expansion in habitat suitability. Habitat suitability is given as a relative scale from lighter color (low suitability) to greener color (high suitability) for projections into the last interglacial (LIG), last glacial maximum (LGM), mid-Holocene, and contemporary climate space.

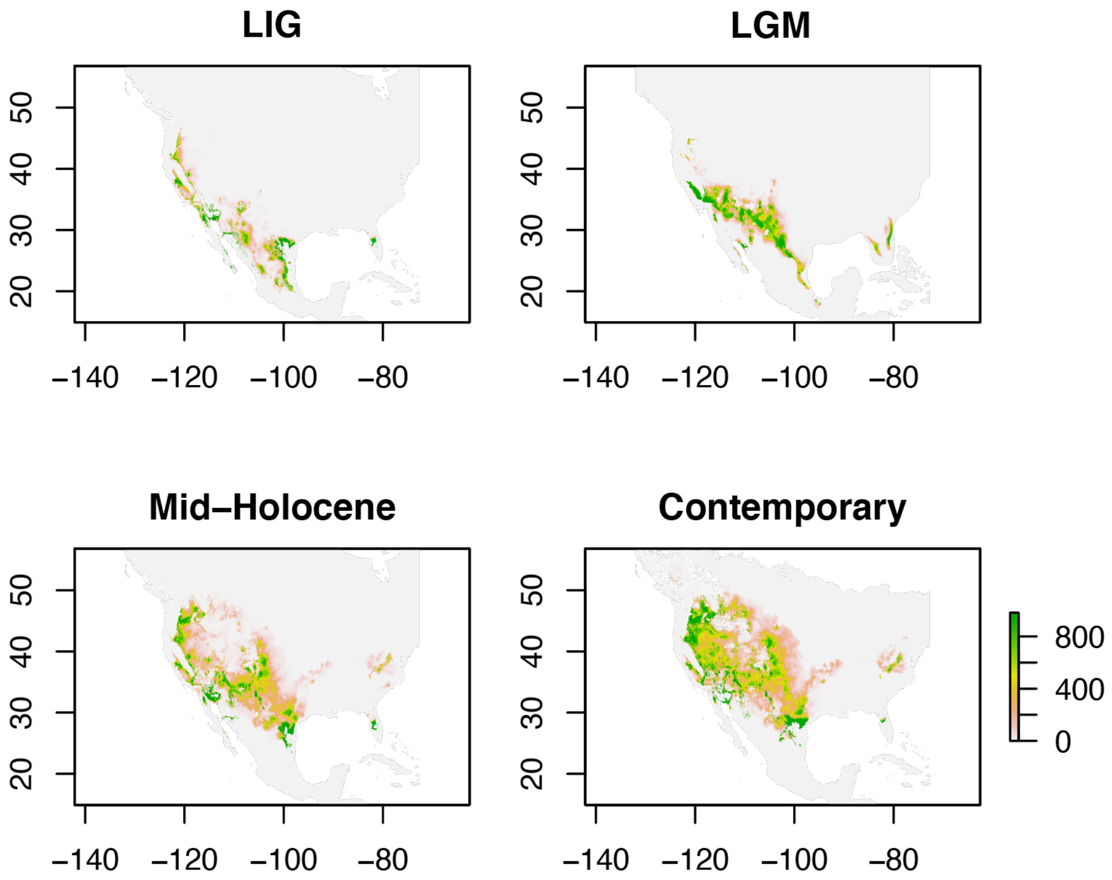

S1 Table 3. ENM run evaluations from full Maxent runs of three background point (PA) datasets. Each dataset of background points are separate Maxent models.

Run	Pseudoabsence-set	Index	Cutoff	Value	Sensitivity	Specificity
Full	PA1	ROC	580.5	0.831	63.158	91.667
Full	PA1	TSS	580	0.548	63.158	91.667
Full	PA1	KAPPA	580	0.569	63.158	91.667
Full	PA2	ROC	15.5	0.564	15.789	98.333
Full	PA2	TSS	15	0.141	15.789	98.333
Full	PA2	KAPPA	15	0.193	15.789	98.333
Full	PA3	ROC	469	0.814	76.316	72.5
Full	PA3	TSS	467.5	0.488	76.316	72.5
Full	PA3	KAPPA	622	0.477	52.632	91.667

S1 Table 4. Predictor importance from three models that were provided different background point datasets (PA).

MAXENT Full (10-eval) Pseudo-absence datasets					
**Predictor**	**PA1**	**PA2**	**PA3**	**Mean**	**SD**
MeanAnnualTemp (bio1)	0.33	0.40	0.20	0.31	0.10
PrecipColdestQuarter (bio19)	0.08	0.28	0.19	0.18	0.10
MeanDiurnal (bio2)	0.32	0.91	0.26	0.49	0.36
Isothermality (bio3)	0.72	0.50	0.58	0.60	0.11
