# Supplementary material for: Once upon a time in Mexico: Holocene biogeography of the spotted bat (Euderma maculatum)
Source: PLoS One. 2023 May 10;18(5):e0274342. doi: 10.1371/journal.pone.0274342 (PMC10171611; doi:10.1371/journal.pone.0274342)

S2 Fig 1. Phylogenetic trees (substitutions/site) of *Euderma maculatum* showing incongruent topologies among three programs and two optimality criteria (RaxML-NG = maximum likelihood; BEAST2 and MrBayes = Bayesian posterior probability). Trees were rooted using *Idionycteris phyllotis* (IDPH) as an outgroup. Colors represent broad, discretized portions of the *E. maculatum* range.

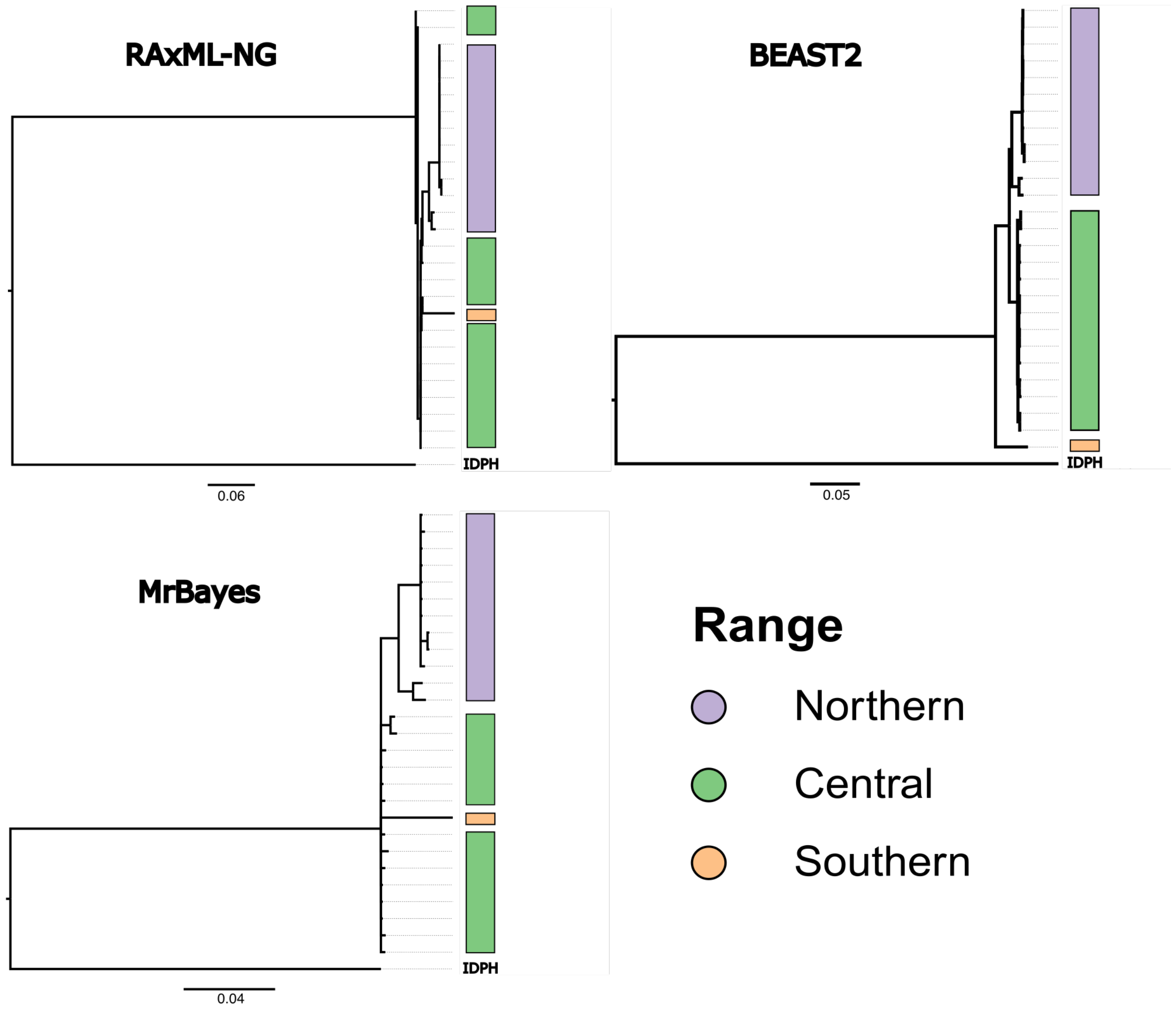

Supplement: S2 File — (PDF) [file pone.0274342.s002.pdf]
